# Intraoperative fluid management in hepato-biliary-pancreatic operation using stroke volume variation monitoring: A single-center, open-label, randomized pilot study

**DOI:** 10.1097/MD.0000000000023617

**Published:** 2020-12-11

**Authors:** Yudai Iwasaki, Yuko Ono, Ryota Inokuchi, Tokiya Ishida, Yoshibumi Kumada, Kazuaki Shinohara

**Affiliations:** a Department of Anesthesiology and Emergency Medicine, Ohta Nishinouchi Hospital; b Emergency and Critical Care Medical Centre, Fukushima Medical University, Fukushima; c Department of Emergency and Critical Care Medicine, JR General Hospital, Tokyo, Japan.

**Keywords:** fluid management, hepato-biliary-pancreatic surgery, intraoperative fluid balance, perioperative care, stroke volume variation

## Abstract

**Trial design:**

This investigator-initiated, single-center, open-label, parallel-group, randomized-controlled pilot study was designed to compare the intraoperative fluid balance and perioperative complications in patients undergoing hepato-biliary-pancreatic surgery with or without stroke volume variation (SVV)-guided fluid management.

**Methods:**

Patients who were aged >18 years and underwent elective major hepato-biliary-pancreatic surgery between June 30, 2015, and August 31, 2016 at our center were randomly assigned to receive SVV-guided or conventional fluid therapy. The intervention group used SVV to determine the patients’ volume status. The primary outcome was the total fluid balance per body weight per operation time, and the secondary outcomes were the total amount of intravenous infusion per body weight per operation time and the Sequential Organ Failure Assessment score on postoperative day 1. Patients were randomized by a two-block computer-generated assignment sequence. Masking of patients and assessors was conducted. The patients and assessors were each blinded to the details of the trial; however, the clinicians were not.

**Results:**

Of the 69 patients who were initially eligible, 60 provided informed consent for participation in the study. After randomization, three patients dropped out of the study because of deviations from the protocol or unexpected hypotension, leaving 28 and 29 patients in the intervention and control groups, respectively. Patients in both groups had similar characteristics at baseline. The median (interquartile range [IQR]) intraoperative fluid balance in the control and SVV groups was 6.2 (IQR, 4.9–7.9) and 8.1 (IQR, 5.7–10.5) ml/kg/h, respectively (*P* = .103). The administered intravenous infusion was significantly higher in the SVV group (median, 10.9; IQR, 8.3–15.3 ml/kg/h) than in the control group (median, 9.5; IQR, 7.7–10.3 ml/kg/h) (*P* = .011). On postoperative day 1, the PaO_2_/FiO_2_ ratio was lower in the SVV group (median, 266; IQR, 261–341) than in the control group (median, 346; IQR, 299–380) (*P* = .019).

**Conclusions:**

Use of the SVV-guided fluid management protocol did not reduce intraoperative fluid balance but increased the intraoperative fluid administration and might worsen postoperative oxygenation.

**Trial registration:**

UMIN000018111.

## 1 Introduction

Fluid administration in the intraoperative period is one of the major responsibilities of anesthesiologists. As fluid overload and restrictive fluid regimens in the intraoperative period can lead to increased morbidity and mortality rates,^[1–5]^ recent guidelines have strongly recommended euvolemic fluid management.^[6]^ Considering that the major abdominal surgeries are associated with pulmonary edema, loss of digestive tract function, and prolonged hospital stay,^[7]^ the use of advanced hemodynamic monitoring during these surgeries is recommended to facilitate such individualized fluid therapy and optimize oxygen delivery.^[6]^

Monitoring static variables, such as central venous pressure (CVP), for perioperative fluid management in the field of hepato-biliary-pancreatic operations could not sufficiently predict patients’ fluid responsiveness.^[8]^ Stroke volume variation (SVV) is a hemodynamic variable that could predict a patient's fluid responsiveness reliably and objectively.^[9–11]^ Past studies have shown an association between goal-directed fluid management using SVV and improved outcomes in patients who underwent highly invasive interventions, such as cardiac, major general, gynecological, and urological surgeries.^[12,13]^ Although a systematic review of the enhanced recovery programs in hepato-biliary-pancreatic surgeries did not mention the importance of fluid administration in the intraoperative period, such significance has been highlighted in the field of colorectal surgeries.^[6,14]^ There are limited data on the effect of goal-directed fluid management using SVV in hepato-biliary-pancreatic subsets, and the previous results are conflicting.^[15,16]^

Moreover, previous studies that have evaluated goal-directed therapy had 2 limitations. First, they used protocols on the comparison group, which may have increased the difference between the two groups. One study used the protocol determined for the control group,^[3]^ which did not correspond with our usual fluid strategy, whereas other studies used CVP and urine output for the intervention of the fluid bolus in the control group.^[2,10,12]^ Marik et al revealed that CVP was not associated with fluid responsiveness,^[17]^ while guidelines recommend that urine output should not be used as the target of fluid administration.^[18]^ Due to these unfavorable settings, the control group may have been administered a large amount of fluid, as the protocol and goal-directed therapy using SVV seemed to be superior. Second, previous studies have set the total fluid volume as the primary outcome. When considering the fluid status validity, patients’ body weight, operation time, and amount of bleeding should also be considered, as they can affect the intraoperative fluid volume. We believe that it is important to consider adding these parameters when assessing the intraoperative fluid volume.

Therefore, the aim of our prospective randomized study was to examine the effects of SVV-guided fluid management on intraoperative fluid balance, and its influence on postoperative organ functions compared to the conventional fluid management methods when performing major hepato-biliary-pancreatic surgeries.

## 2 Materials and methods

### 2.1 Study design and setting

This was an investigator-initiated, single-center, open-label, parallel-group, randomized-controlled clinical trial conducted at the Ohta Nishinouchi teaching and referral Hospital, Fukushima, Japan. The annual number of surgeries performed in this facility is approximately 5,200. Of these, approximately 2% are operations involving hepato-biliary-pancreatic resections. The study protocol and statistical analysis plan were approved by the Institutional Review Board of the Hospital (No. 85-1, May 26, 2015) and performed in accordance with the Declaration of Helsinki. The trial was registered at the University Hospital Medical Information Network (UMIN) (Registration no. UMIN000018111, July 26, 2015). An independent data and safety monitoring committee consisting of in-house experienced anesthesiologists, surgeons, and emergency physicians supervised the study and reviewed the blinded data. There was no industry support or involvement in this clinical trial. Patients were screened and randomly assigned to the SVV or conventional group between June 30, 2015, and August 31, 2016. Written informed consent was obtained from each patient prior to randomization. Only the participating anesthesiologists who collected the data during surgery were aware of the group assignments. The patients, researcher who performed statistical analysis, members of the safety monitoring committee, data abstracters, secretaries who entered data into spreadsheets, and surgeon who performed the postoperative managements were blinded to these group assignments.

### 2.2 Participants and randomization

Patients aged >18 years who were scheduled to be admitted to the intensive care unit (ICU) after an elective major hepato-biliary-pancreatic operation were included in this study. Patients were excluded if they had undergone only cholecystectomy. Those who experienced atrial fibrillation, had anemia (hemoglobin <7 g/dl) or hypoalbuminemia (serum albumin <2.0 g/dl), or were on maintenance hemodialysis were also excluded, as they were more likely to receive early transfusion, albumin, and vasoconstrictors in the perioperative care period.^[19–21]^ Patients were also excluded from analysis when active resuscitation was required due to massive bleeding or when anaphylaxis or cardiac arrest was anticipated.^[22]^ Randomization was performed using a two-block computer-generated assignment sequence by the primary investigator. Patient assignments were determined by the senior anesthesiologists who adopted a supervisory role.

### 2.3 Anesthesia procedures

In patients who had no coagulopathy and had not received any antiplatelet or anticoagulant drugs, an epidural catheter was placed between T7 and T10 before the induction of general anesthesia. A 16-G or 18-G intravenous catheter and a 22-G intra-arterial catheter were placed in the patient's forearm, and a triple lumen central venous catheter (CVC) was inserted into the right internal jugular vein, when required. General anesthesia was induced using intravenous propofol (1.0–2.0 mg/kg), remifentanil (0.25–0.5 μg/kg/min), and rocuronium (>0.6 mg/kg). The patient's trachea was intubated with a single lumen endotracheal tube at the appropriate depth. Then, mechanical ventilation was started with a tidal volume of 8 ml/kg of ideal body weight, with positive end-expiratory pressure of 5 cm of water. The ideal body weights of male and female participants were 50.0 ± 0.91 (height, 152.4 cm) and 45.5 ± 0.91 (height, 152.4 cm) kg, respectively. Anesthesia was maintained with sevoflurane inhalation (1–1.5%), continuous intravenous administration of remifentanil (0.1–0.5 μg/kg/min) and rocuronium (10 μg/kg/min for 1 hour followed by 7 μg/kg/min), and an intravenous bolus of fentanyl.

Basic anesthetic monitoring devices, including an electrocardiogram, a percutaneous oxygen saturation monitor, non-invasive and invasive blood pressure monitors, and capnometry were used throughout the operations. Postoperative analgesia was provided through the epidural infusion of levobupivacaine or intravenous infusion of fentanyl. Epidural infusion was not administered until the initiation of abdominal wall closure, as it could affect the total fluid balance intraoperatively. With the exception of the interventions described below, all other treatments were conducted at the discretion of the participating anesthesiologists.

### 2.4 Interventions and control group

**Figure 1:**
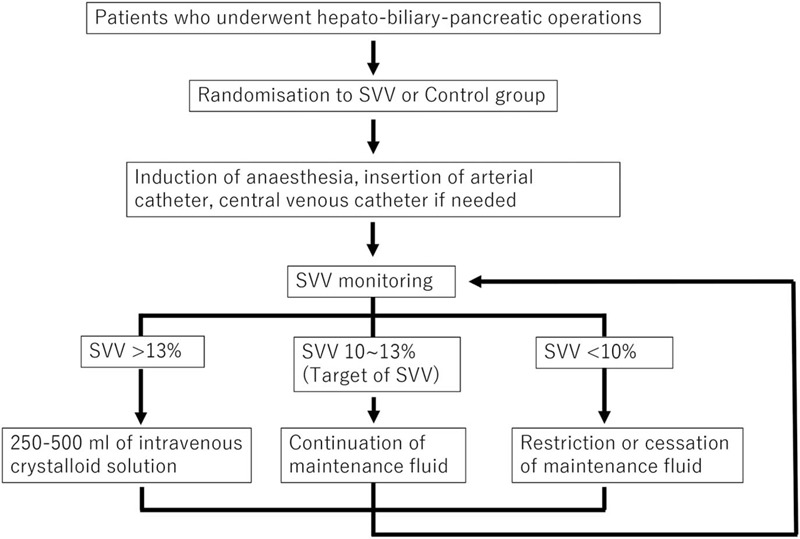
Protocol of intraoperative infusion strategy during hepato-biliary-pancreatic surgeries. SVV = stroke volume variation.

Patients in the intervention group received intravenous fluid and blood transfusion according to the SVV-guided hemodynamic therapy protocol. To monitor SVV, a FloTrac sensor (EV1000, Edwards Lifescience, Irvine, CA) was used. The SVV was maintained between 10% and 13% in accordance with the manufacturer's recommendation and previous researches.^[9,22,23]^ Anesthesiologists recorded the SVV value every 15 minutes. A total of 250 to 500 ml of intravenous crystalloid solution was administered to achieve and maintain the target SVV. When the SVV dropped to <10%, indicating that the patient was in a hypervolemic state, the administration of maintenance crystalloid was restricted or stopped until the SVV increased to >10% (Fig. 1). The choice of crystalloid was at the discretion of the attending anesthesiologist, while the use of colloids and transfusions was at the discretion of the treating anesthesiologists and surgeons.

When the SVV reached the targeted score, phenylephrine (0.05 mg) or ephedrine (4 mg) was administered intravenously to maintain a mean arterial blood pressure (MAP) of 65 mmHg or a systolic blood pressure of 90 mmHg. The conventional care group received the usual perioperative care and the MAP of patients in this group was maintained at 65 mmHg (via volume overload or catecholamine use). However, a dynamic hemodynamic monitor was not used in this group.

### 2.5 Data acquisition

Data on the duration of operation; amount of ephedrine, phenylephrine, and fluids (including crystalloids, colloids, and transfusion) administered; urine output; blood loss; and the SVV values recorded every 15 minutes were collected from the anesthesia records. Data from the electronic medical records included patients’ height and weight, past medical history, MAP and oxygen saturation after being admitted to the ICU, and results of blood tests from postoperative day 1 (POD1), such as platelet count, bilirubin, PaO_2_, and creatinine levels.

### 2.6 Outcomes

The primary outcome in this study was total fluid balance per body weight per operation time (in-out/kg/h). Although some studies have set the total amount of fluid volume as the primary outcome,^[10,13]^ fluid administration depends on the length of the operation and the patient's body weight. Therefore, in this study, we employed fluid balance, measured as in-out/kg/h, as the primary outcome. The secondary outcomes were the total amount of intravenous infusion per body weight per operation time (in/kg/h) and the Sequential Organ Failure Assessment (SOFA) score on POD1 by measuring the function of the respiratory, cardiovascular, hepatic, coagulation, renal, and neurological systems. When PaO_2_ measurement on POD1 was not available, the PaO_2_/FiO_2_ ratio was calculated from the value for the pulse oximetric saturation/FiO_2_ ratio using a formula.^[24]^ The participating anesthesiologists and surgeons who provided postoperative care were blinded to the outcome assessment plan to mitigate postoperative therapeutic bias. For the same reason, the investigator who assigned patients to groups (author YI) was not involved in any of the clinical practices.

### 2.7 Sample size

While planning this study, a power analysis was performed using G∗Power 3 for Windows (Heinrich Heine University, Dusseldorf, Germany). Based on our retrospective preliminary data (20 patients), total fluid balances of 9.5 (standard deviation [SD], 4.4) and 6.5 (SD, 1.5) usual care, respectively. From these data, effect size *d* was calculated as 0.82. When effect size *d* was set as 0.8 with 80% power at a two-tailed α of .05, we estimated that a sample size of 26 patients in each group would be needed to detect a 28% difference in the primary outcome. We set the sample size as 60 to accommodate the 5% of patients that had dropped out, which met the requirement of the pilot study's sample size.^[25]^

### 2.8 Statistical analysis

Analyses were performed according to an a priori statistical analysis plan including all patients on a per protocol analysis. Differences in continuous variables, including age, body weight, body mass index (BMI), operation time (h), total fluid balance (in-out/kg/h), and total amount of intravenous infusion (in/kg/h), between the 2 groups were compared using Student's *t* test, following verification of the data normal distribution using the Shapiro-Wilk test. When the data were not normally distributed, the Mann–Whitney *U* test was used. Differences in the ordinal scale, including the American Society of Anesthesiologist's physical status (ASA-PS) classification, and the SOFA scores between the 2 groups were compared using the Mann–Whitney *U* test. Differences in categorical variables, including sex, mortality, and indication for epidural anesthesia, between the two groups were compared using Fisher exact test. All statistical analyses were performed using SPSS Statistics for Windows, version 22.0 (IBM Corp., Armonk, NY). A *P* value <.05 was considered statistically significant.

## 3 Results

**Figure 2:**
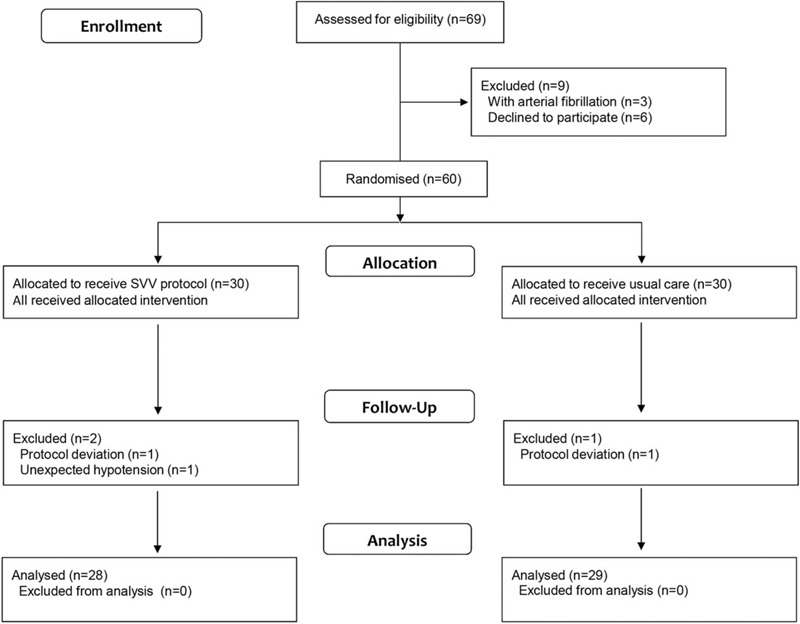
Assessment, randomization, and follow-up framework for patients included in this study.

A total of 69 patients were eligible to participate in this study. After assessment for the inclusion criteria and obtaining informed consent, 60 patients were included and randomly assigned to receive the SVV protocol or usual care treatment. Three patients dropped out after randomization because of deviations from the protocol or unexpected hypotension (2 and 1 patients from the SVV protocol and the control group, respectively). There were no other discontinuations or patients lost to follow-up (Fig. 2). Thus, 28 and 29 patients were included in the intervention and control groups, respectively.

Both groups had similar basic characteristic parameters including age, sex, BMI, ASA-PS classification score, surgical procedure, and use of epidural anesthesia. No significant differences in the proportion of comorbidities were observed (Table 1). Table 2 shows the perioperative data for each group. Duration of starvation, operation, ICU stay, and mechanical ventilation were similar between the 2 groups. In the intervention group, the SVV was maintained at approximately 11%. The fluid balance (in-out/kg/h) did not significantly differ between the groups (control group: median, 6.2; IQR, 4.9–7.9 ml/kg/h; intervention group: median, 8.1; IQR, 5.7–10.5 ml/kg/h; *P* = .103). However, in terms of in/kg/h, the SVV protocol group had a significantly higher fluid balance (control group: median, 9.5; IQR, 7.7–10.3 ml/kg/h; intervention group: median, 10.9; IQR, 8.3–15.3 ml/kg/h; *P* = .011).

**Table 1:** Baseline characteristic of patients enrolled in this study^∗,†^.

	Control (n = 29)	Intervention (n = 28)
Age (years)	71 (64–76)	67 (61–76)
Actual body weight (kg)	59.4 (56.8–66.5)	57.8 (49.8–69.3)
Height (cm)	162.7 (158.8–166)	162 (153.8–168)
Body mass index (kg/m^2^)	22.9 (21.5–24.8)	23.0 (20.4–25.1)
Ideal body weight (kg)	59.4 (55.8–62.4)	58.7 (51.2–64.2)
Male, n (%)	25 (86)	22 (79)
Hypertension, n (%)	21 (74)	13 (46)
Coronary disease, n (%)	3 (10)	0 (0)
Chronic heart failure, n (%)	0 (0)	4 (14)
Stroke, n (%)	2 (7)	4 (14)
Chronic obstructive pulmonary disease n (%)	3 (10)	7 (25)
Diabetes, n (%)	13 (45)	9 (32)
Chronic kidney disease, n (%)	4 (14)	0 (0)
Cancer, n (%)	29 (100)	26 (93)
ASA-PS ≧ 3 (%)	22 (76)	22 (76)
Operative data		
Hepatic resection, n (%)	20 (69)	15 (54)
Pancreatic resection, n (%)	8 (28)	12 (43)
Others, n (%)	1 (3)	1 (4)
Insertion of epidural catheter, n (%)	23 (79)	23 (82)
28-day mortality n (%)	0 (0)	0 (0)

^∗,†^ Values are presented as median (interquartile range) or number (%). ASA-PS = American Society of Anesthesiologists – physical status.

**Table 2:** Perioperative patient information^∗^.

	Control (n = 29)	Intervention (n = 28)	*P* value
Duration of starvation (h)	3 (3.0–3.0)	3 (2.8–3.0)	.553
Duration of operation (h)	5.6 (3.6–7.7)	4.7 (3.6–6.8)	.621
Amount of ephedrine (mg)	0 (0–8)	6.5 (0–16)	.33
Amount of phenylephrine (mg)	0 (0–0.1)	0 (0–0.1)	.86
Crystalloids (ml)	2200 (1700–2600)	2025 (1483–3253)	.943
Colloids (ml)	500 (200–1000)	500 (0–750)	.468
Transfusion (ml)	0 (0–0)	0 (0–770)	.06
Output total (ml)	730 (630–1190)	944 (664–1676)	.247
Blood loss (ml)	500 (400–734)	625 (395–1105)	.271
Urine output (ml)	230 (160–332)	252 (118–370)	.792
Fluid balance (ml)	2060 (1395–2700)	2169 (1645–2988)	.425
Fluid balance/kg/h (ml/kg/h)	6.2 (4.9–7.9)	8.1 (5.7–10.5)	.103
In total/kg/h (ml/kg/h)	9.5 (7.7–10.3)	10.9 (8.3–15.3)	.028
In total/IBW/h (ml/kg/h)	9.8 (8.0–10.5)	11.7 (9.1–14.6)	.011
Duration of ICU (days)	2 (2–3)	2 (2–2)	.105
Duration of mechanical ventilation (days)	0 (0–0)	0 (0–0)	.606
Mean SVV (%)		10.7 (9.775–12.025)	

∗ Data are expressed as median (interquartile range) unless otherwise indicated. IBW = ideal body weight, ICU = Intensive care unit, SVV = stroke volume variation.

Postoperative data for each group are summarized in Table 3. There was no difference in the SOFA score between the groups on POD1. The PaO_2_/FiO_2_ ratio was lower in the SVV protocol group (control group: median, 346; IQR, 299–380; intervention group: median, 266; IQR, 261–341; *P* = .019). There were no important harms in the two groups.

**Table 3:** Postoperative day 1 sequential organ failure assessment scores.

SOFA POD1^∗^	Control (n = 29)	Intervention (n = 28)	*P* value
PaO_2_/FiO_2_ ratio	346 (299–380)	266 (261–341)	.019
Platelet count (^∗^10τ/ml)	12.4 (9.5–16.4)	12.3 (9.0–16.5)	.867
Bilirubin (mg/dl)	1.6 (1.3–2.2)	1.7 (1.1–2.6)	.861
Minimum MAP (mmHg)^†^	61 (13)	59 (15)	.582
GCS	15 (15–15)	15 (15–15)	.146
Creatinine (mg/dl)	0.8 (0.6–1.0)	0.7 (0.5– 0.8)	.267
SOFA score on POD1	4 (3–5)	4 (3–5)	.674

GCS = Glasgow coma scale, MAP = mean arterial pressure, PaO_2_/FiO_2_ = partial pressure of arterial oxygen/fraction of inspiratory oxygen, POD1 = postoperative day 1, SOFA = sequential organ failure assessment.

∗ Values are presented as median (interquartile range) unless otherwise indicated.

† mean (standard deviation).

## 4 Discussion

The principal finding of this clinical trial was that the SVV-guided goal-directed therapy algorithm was not associated with a significant reduction in the intraoperative fluid balance (in-out/kg/h) in patients who underwent major hepato-biliary-pancreatic surgery compared to that in patients who received the usual care. In fact, we observed that the SVV-guided management resulted in increased intraoperative fluid administration (in/kg/h). In this trial, we did not find any difference in the secondary outcomes of the SOFA scores on POD1 between the groups. However, patients who received the SVV-guided goal-directed therapy had worse PaO_2_/FiO_2_ ratios, which, among other causes, might have been the result of pulmonary edema due to increased intraoperative fluid administration. Our findings suggested that SVV-guided goal-directed therapy should be employed cautiously in patients requiring major hepatobiliary pancreatic surgery. Our study also suggested the importance of weight-adjusted fluid assessment and associated clinical outcomes, as Gottin et al revealed the usefulness of developing weight-adjusted fluid strategies.^[26]^ In their study, patients who received goal-directed therapy could not reduce the fluid amount compared with those receiving restricted fixed-volume weight-guided fluid management (4 ml/kg/h).

The reported effectiveness of monitoring SVV for fluid restriction has been controversial. Hofer et al. reported that SVV was a good predictor of fluid responsiveness in patients undergoing coronary artery bypass grafting.^[11]^ Jan et al and Erik et al reported regarding the efficacy of SVV monitoring during abdominal surgery.^[10,16]^ In contrast, Lahner et al emphasized that SVV was not associated with fluid responsiveness in patients undergoing major abdominal surgery.^[15]^ We did not achieve fluid restriction using the SVV-guided protocol, and we could not find any advantages with respect to postoperative organ dysfunction. One possible reason for this discrepancy could be the identification of in-out/kg/h, as the primary outcome. Most previous studies have evaluated the total in-out balance of fluid for each patient, omitting the fact that the total fluid balance depends on the body weight of patients and the operation length. Evaluating the outcome using in-out/kg/h may have resulted in a more thorough evaluation, suggesting that an SVV protocol for fluid restriction in hepatobiliary surgical procedures is ineffective.

The second plausible explanation for these discrepancies is that in our protocol we did not use catecholamines continuously to maintain the MAP, as per the SVV-guided protocol. According to a previous study, hypotension during surgery is not related to poor postoperative outcomes.^[27]^ Therefore, to maintain MAP, we administered a bolus of ephedrine or phenylephrine based on the judgment of the responsible anesthesiologist. Interestingly, continuous administration of catecholamines could increase the stressed volume of the venous circulation by lowering venous compliance.^[28]^ A recent study reported the use of a goal-directed therapy protocol for perioperative fluid management, which included the use of norepinephrine according to the MAP.^[29]^ Without continued catecholamine use in this study, the volume of perioperative fluid administered in each patient might have increased.

On another note, the target SVV value may have been set higher in our study. While many studies aimed to achieve an SVV of approximately 10%, a prior study set the target for SVV to 20% for perioperative fluid management of patients undergoing pancreatoduodenectomy.^[30]^ Setting a high target for SVV might improve fluid restriction and postoperative prognosis. Moreover, the anesthesiologists might have used a more restrictive regimen in the control group based on their clinical experience.

### 4.1 Limitations

This study had several limitations. First, as the participating anesthesiologists could not be blinded to the intervention, there was a possibility of intraoperative therapeutic bias. To minimize this bias, details of our outcome assessment plans were masked from the participating anesthesiologists. Moreover, the investigator who assigned the patient groups (author YI) was not involved in any clinical practice after surgery. To mitigate postoperative therapeutic bias, the surgeons providing postoperative care were also blinded to the study assignments and outcomes. As all hemodynamic variables were measured using a FloTrac device, an ascertainment bias was less likely, as the collected values were reliable and less prone to measurement errors. As this was a single-center trial, the main researcher had to be involved in the allocation of patients, which might have led to possible selection bias. Second, our study was conducted in a single community hospital in Japan. Therefore, the results may not be generalized to other settings. This study also targeted patients who underwent regular hepatobiliary pancreatic surgery. Therefore, the findings cannot be generalized to populations not included in the trial, such as patients who underwent other major abdominal or emergency surgeries. Finally, we did not measure systemic vascular resistance (SVR), which might be an alternative variable for venous compliance. For meticulous assessment, measurement of SVR is also important. However, to minimize patient invasiveness, insertion of CVC was not mandatory in our study protocol.

### 4.2 Future directions

Despite these limitations, this study also had several strengths. To the best of our knowledge, this was the first randomized clinical trial to assess the effect of SVV-guided goal-directed therapy in patients undergoing major hepato-biliary-pancreatic surgery using in-out/kg/h as the primary outcome. The results of our study suggested that more prudent interpretation is required when using this approach in this population. Some previous studies have recommended the SVV-guided protocol for the abdominal surgeries. However, our study assessed fluid volume meticulously, and we revealed that the SVV-guided protocol, which targeted SVV for 10% to 13%, did not have any advantages compared with usual fluid management. Moreover, the SVV-guided protocol was assessed by comparing with usual care strategy, which did not use CVP and urine output, which are inappropriate surrogate targets of fluid responsiveness. Our study provided the rationale for future clinical trials to better clarify the role of SVV-guided fluid management in the hepato-biliary-pancreatic operations. In the future, a large, randomized control study should be conducted to compare the outcomes derived from usual fluid and goal-directed therapies in patients undergoing the operations.

In conclusion, the use of the SVV-guided goal-directed therapy algorithm did not reduce the intraoperative fluid balance. Rather, it increased the intraoperative fluid administration volume and worsened oxygenation on POD1 in our participants who underwent hepato-biliary-pancreatic surgery. Our results showed the potential pitfall of SVV-guided goal-directed therapy for patients requiring highly invasive hepato-biliary-pancreatic surgeries; thus, when SVV is utilized for fluid management, the SVV target values might have to be set higher.

## Acknowledgments

We thank our colleagues at the Department of Anesthesiology and Surgery at Ohta Nishinouchi Hospital for data acquisition and for their contribution to this study.

## Author contributions

**Conceptualization:** Yudai Iwasaki, Yuko Ono, Ryota Inokuchi

**Data curation:** Yudai Iwasaki, Yuko Ono

**Formal analysis:** Yudai Iwasaki

**Investigation:** Yudai Iwasaki

**Methodology:** Yuko Ono, Ryota Inokuchi, Tokiya Ishida

**Supervision:** Yuko Ono, Ryota Inokuchi, Tokiya Ishida, Yoshibumi Kumada, Kazuaki Shinohara

**Validation:** Tokiya Ishida

**Writing – original draft:** Yudai Iwasaki, Yuko Ono, Ryota Inokuchi, Tokiya Ishida

**Writing – review & editing:** Yuko Ono, Ryota Inokuchi, Tokiya Ishida, Yoshibumi Kumada, Kazuaki Shinohara
